# The phosphoenolpyruvate phosphotransferase system regulates cell length and width through semi-independent pathways

**DOI:** 10.1091/mbc.E26-03-0129

**Published:** 2026-07-27

**Authors:** Jacob Surber, Randy M. Morgenstein

**Affiliations:** ^a^Department of Microbiology and Molecular Genetics, Oklahoma State University, 307 Life Science East, Stillwater, OK 74078; Johns Hopkins University

## Abstract

It has been known for over 80 years that bacterial cell size is affected by growth conditions. Cells grown in a nutrient-rich medium are larger and wider than those grown in a nutrient-poor medium. Yet even after decades of research, it is still not fully known how metabolism and cell size are coregulated. In this work, we describe a new source of metabolic control over *Escherichia coli* cell size, the phosphoenolpyruvate phosphotransferase system (PTS). The PTS is used to phosphorylate sugars upon entry into the cell. We found that mutations in this system result in both shorter and thinner cells and that the regulation of both dimensions of cell size appears to come from two separate mechanisms. The first mechanism regulates cell length through the production of cAMP, while the second mechanism regulates cell width through control of the levels of PEP or pyruvate in the cell.

## Abstract (executive summary)

- A fundamental question in cell biology is how cells achieve their size and shape.
- Bacterial size is known to be regulated by the metabolic conditions in which the cells are grown, although all the ways metabolism and cell size are mechanistically connected are unknown.
- Here we found that the sugar import system, the phosphoenolpyruvate phosphotransferase system (PTS), regulates both cell width and cell length even in the absence of sugar in the growth media. The PTS can regulate levels of both cyclic-AMP and pyruvate in the cell, which in turn regulates the length and width of the cell.

## INTRODUCTION

Cells tightly regulate their size when grown in a given growth condition. When grown in a nutrient-rich medium, bacterial cells grow faster and are longer and wider than when they are grown in a nutrient-poor medium (Schaechter *et al.*, 1958). This phenomenon has been termed the “Growth Law” and relates to growth in different nutrient conditions, as changes in growth rate due to temperature changes do not have the same effect on cell size. While the “Growth Law” has been known for almost 80 years, the molecular basis for connecting cell size to nutrient availability has yet to be fully determined. The alarmone, ppGpp, which is produced during nutrient starvation, leads to a reduction in cell size (Schreiber *et al.*, 1995). Furthermore, the production of fatty acids has been shown to help regulate cell size (Yao *et al.*, 2012; Vadia *et al.*, 2017). In addition, the molecule UDP-glucose has been shown to act as an intermediary between carbon availability and cell length by regulating the main division protein, FtsZ, in both *Escherichia coli* and *Bacillus subtilis* (Chien *et al*., 2012; Hill *et al*., 2013; Weart *et al*., 2007). In *B. subtilis,* the levels of the central carbon metabolite pyruvate were shown to suppress a defective version of FtsZ (Monahan *et al.*, 2014). Interestingly, pyruvate is a precursor to the building blocks of fatty acids, further linking regulation of cell division with the metabolic state of the cell (for a more detailed explanation of metabolism and cell size, see reviews; Sperber and Herman, 2017; Westfall and Levin, 2017).

**FIGURE 1:**
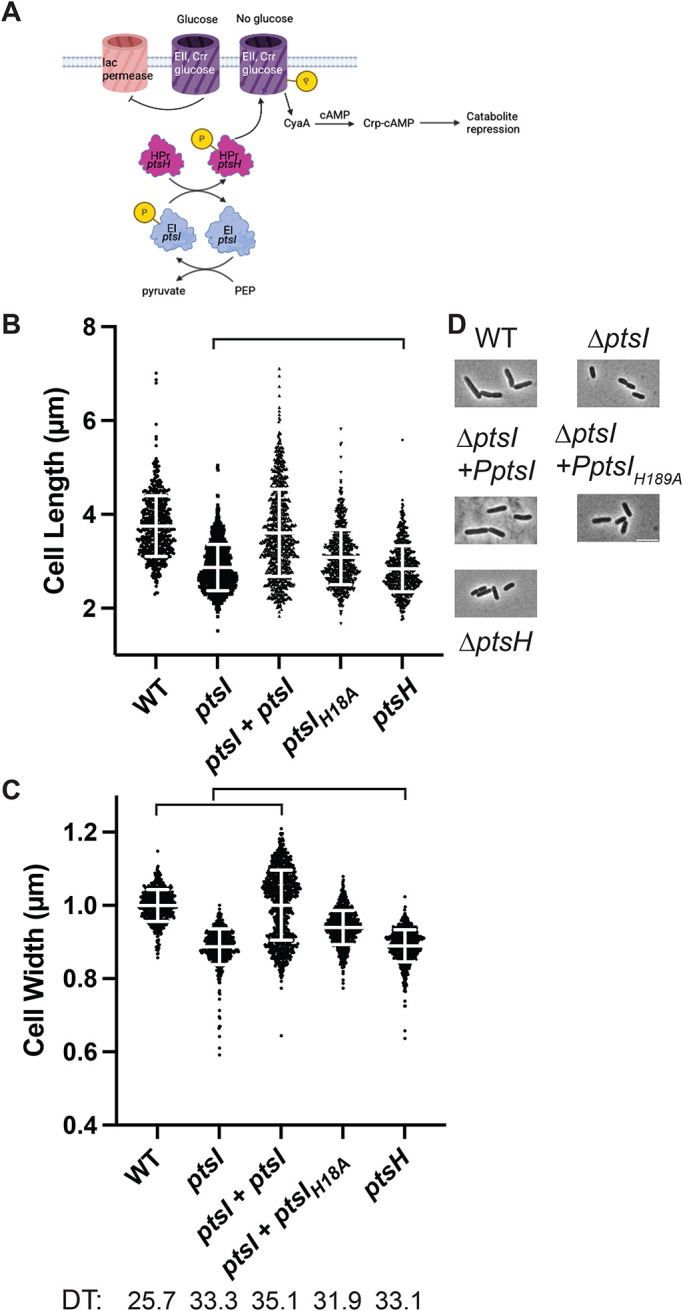
The PTS regulates both cell length and width. A) Model depicting the role of PTS proteins. Created in https://BioRender.com. B–D) Cells were grown in LB media with 0.2% arabinose to mid-log phase. Length and width measurements were taken from single cells of indicated strains. White bars are mean with SD from three replicates pooled together. N: WT = 555, *ptsI* 779, *ptsI + ptsI* = 821, *ptsI + ptsI_H189A_* = 682, and *ptsH* = 648 cells. All comparisons are statistically significant from a one-way Anova and Tukey's multiple comparison test *p* < 0.001. Indicated comparisons are not significant. All strains contain either pBad18 with GFP as a control or indicated *ptsI* allele. DT is the max doubling time. D) Representative images of cells from BC, scale bar = 5 µm.

Using the MreB inhibitor A22, we found that loss of *ptsI,* Enzyme I in the phosphoenolpyruvate phosphotransferase system (PTS) leads to increased resistance to this drug and smaller cells (Sloan *et al.*, 2022). MreB is the major protein involved in cell elongation in most rod-shaped bacteria and has been shown to regulate cell width, suggesting that the PTS may link cell width changes to metabolic state (Ouzounov *et al.*, 2016). The PTS is used to import a variety of sugars into the cell (Deutscher *et al.*, 2014). As sugars, such as glucose, are imported through this system, they are phosphorylated (Buhr *et al.*, 1994). The phosphate for this reaction does not come from the common phosphoryl donor ATP, but rather from phosphoenolpyruvate (PEP). In *E. coli*, the phosphate is removed from PEP by Enzyme I (EI), which passes it to the carrier protein HPr (Figure 1A). While these two proteins are conserved throughout the system, the actual sugar import channel, termed Enzyme II (EII), is unique to each sugar brought into the cell (Tchieu *et al.*, 2001). We have recently reported that deletion of *ptsI*, encoding EI, results in smaller cells, suggesting a new metabolic regulator of cell size in *E. coli* (Sloan *et al.*, 2022).

Not only is the PTS needed for sugar import, but it also regulates catabolite repression by linking glucose levels to cyclic-AMP (cAMP) production (Feucht and Saier, 1980). When glucose levels are low, the entire system is in a phosphorylated state as there is no sugar onto which to pass the phosphate. The phosphorylated glucose-specific EII (c*rr*) can regulate the activity of adenylate cyclase (*cyaA*), the producer of cAMP (Buhr *et al.*, 1994; Feucht and Saier, 1980). During catabolite repression, cAMP binds Crp (cAMP receptor protein) and allows the transcription of alternative catabolic pathways; however, when grown in high glucose levels, the cells rapidly import glucose, leaving EII in an unphosphorylated state, thereby lowering the amount of cAMP and leaving Crp in an inactive state (Görke and Stülke, 2008; Figure 1A).

Here, we report that the reduction in cell size in *E. coli* (length and width) caused by the loss of the PTS is due to two mechanisms. The first pathway results in the majority of the cell shortening and is caused by lower levels of cAMP or CyaA activity. This is hypothesized to be due to the lack of activation of adenylate cyclase caused by the lack of EII phosphorylation. The second pathway regulates the majority of the cell thinning through the change of either PEP or pyruvate levels. EI normally removes a phosphate from PEP, forming pyruvate, but in the absence of EI, this reaction does not take place, potentially changing the levels of either PEP and/or pyruvate in the cell. Taken together, these results add to our understanding of how cells coordinate growth with nutrient availability.

## RESULTS

### A fully functional PTS for glucose import is necessary for proper cell size regulation

We have recently shown that deletion of *ptsI* (Enzyme I, EI) of the PTS results in shorter cells that are more tolerant to high levels of the MreB depolymerizing drug, A22 (Supplemental Figure S1; Sloan *et al.*, 2022). In addition, we found that the ability of EI to be phosphorylated was needed to complement the A22 phenotype (Sloan *et al.*, 2022). To further examine the effects of the PTS on cell size, we measured the length and width of the *ptsI* deletion expressing GFP from pBad18 (as a control) compared with a strain complemented with a mutant allele unable to be phosphorylated (*ptsI*_H189A_) or a wild-type *ptsI* allele (Lopian *et al.*, 2010; Su and Berger, 2012). We found that the lack of *ptsI* or the inability to phosphorylate EI results in cells that are both shorter and thinner than WT cells (Figure 1). While the ability of EI to be phosphorylated is critical for proper cell size regulation, ∆*ptsI* cells complemented with *ptsI*_H189A_ are both longer and wider than cells lacking *ptsI*, suggesting partial complementation. However, these cells are still significantly shorter and thinner than cells complemented with a WT allele of *ptsI.*

To further explore the role of the PTS in cell size regulation, we deleted *ptsH*, the gene encoding HPr, the phosphotransfer protein that accepts the phosphate from EI and transfers it to an EII complex. Similar to the loss of *ptsI*, cells lacking *hpr* are smaller in both dimensions (Figure 1) and more tolerant to A22 than WT cells (Supplemental Figure S1A). This suggests that either a working PTS is needed for size regulation or that phosphorylated HPr has a secondary role in the cell. Both phosphorylated and non-phosphorylated HPr have been shown to interact with and regulate other metabolic genes (Seok *et al.*, 2001; Park *et al.*, 2013; Rodionova *et al.*, 2017). If unphosphorylated HPr is playing a secondary role in cell size regulation, we would have expected to see differences in the ∆*hpr* (no HPr) and *ptsI*_H189A_ (unphosphorylated HPr due to lack of EI phosphorylation) cells. As these strains both exhibit a reduction in cell size and an increase in A22 resistance, HPr is most likely not playing a direct secondary role in cell size regulation (Sloan *et al.*, 2022). However, cells expressing *ptsI*_H189A_ are larger than the ∆*hpr* cells, suggesting a slight role of nonphosphorylated HPr in regulating cell size. This is most likely due to some basal activity of HPr in the PTS.

**FIGURE 2:**
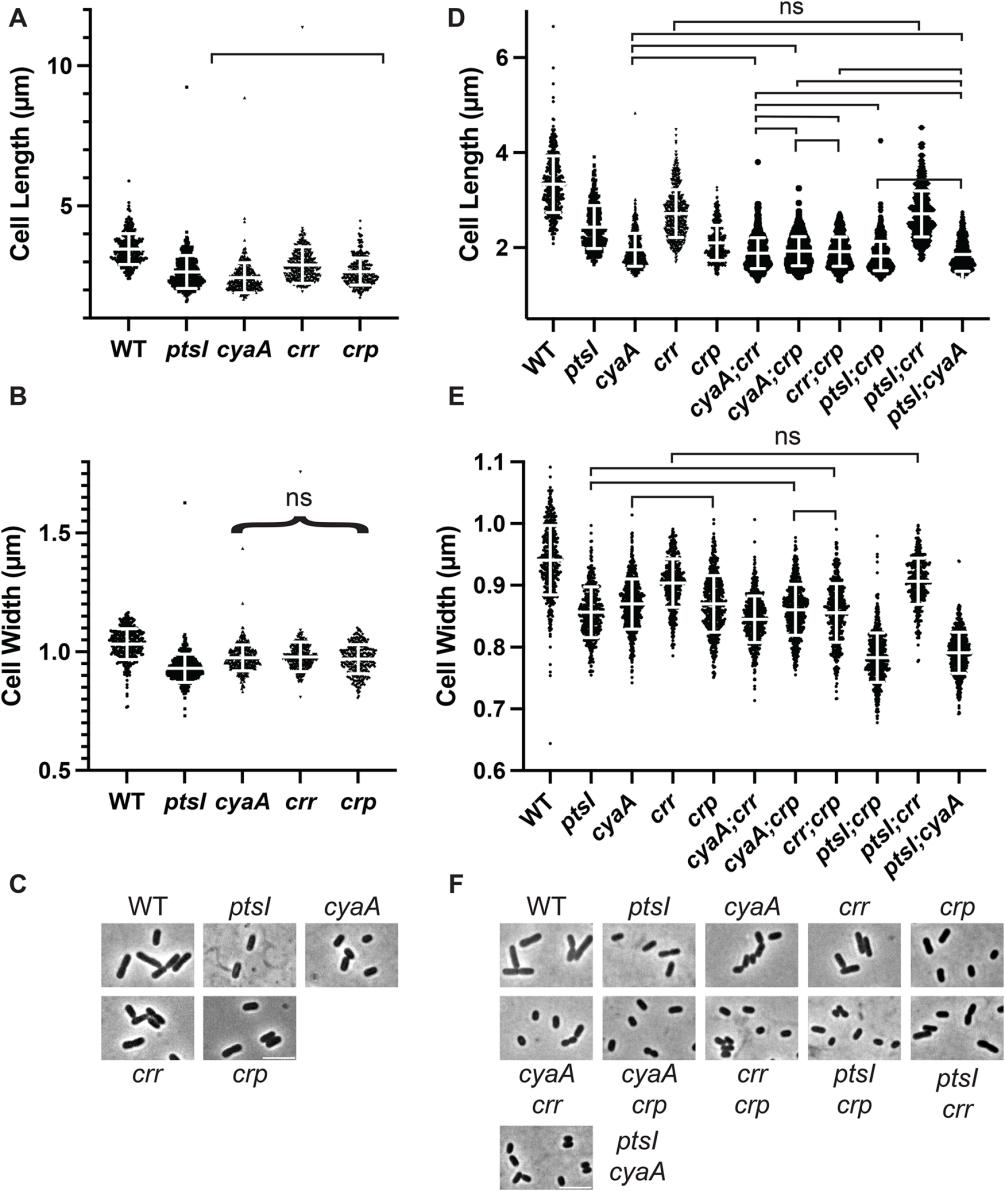
cAMP regulates cell length through two mechanisms. Cells were grown in LB to mid-log phase and length and width measurements of indicated strains were taken. A and B) All comparisons are statistically significant from a one-way Anova and Tukey's multiple comparison test *p* < 0.001. Indicated comparisons are not significant. Indicated comparisons under the bracket are not significant. N: WT = 325, *ptsI* = 397, *cyaA* = 364, *crr* = 358, *crp* = 325 cells. White bars are mean with SD from pooled data of three replicates. C) Representative images of cells from AB, scale bar = 5 µm. D and E) All comparisons are statistically significant from a one-way Anova and Tukey's multiple comparison test *p* < 0.001, unless noted. N: WT = 412, *ptsI* = 416, *cyaA* = 580, *crr* = 461, *crp* = 497, *cyaA;crr* = 549, *cyaA;crp* = 572, *crr;crp* = 468, *ptsI;crp* = 653, *ptsI;crr* = 402, and *ptsI;cyaA* = 608 cells pooled from triplicate experiments. D) *cya – cya;ptsI* and *crr;crp – ptsI;crp* have a *p* < 0.005. E) *ptsI – cyaA;crr*, *cyaA – cyaA;crp, crp – cyaA;crp* and *cyaA;crp – crr;crp* have a *p* < 0.005, and *ptsI;crp – ptsI:cyaA* have a *p* < 0.05. F) Representative images of cells from DE, scale bar = 5 µm.

To further examine if HPr has a secondary role in the cell, we tested the effect of deleting different EII complexes (sugar importers) on changes to cell size. If HPr is acting directly to regulate cell size, deletion of EII complexes should not affect cell size. While there are over 10 different EII complexes in *E. coli* involved in the import of different sugars, we deleted three EII enzymes that we hypothesized might be important for cell wall synthesis due to the sugar they import: *nagE* (N-acetylglucosamine), *chbA* (chitobiose - glucosamine disaccharides), and *murP* (N-acetylmuramic acid; Supplemental Figure S1B). The loss of any of these genes does not result in cells with changes in cell size; however, loss of *crr*, the glucose-specific importer (EII), has been shown to influence cell size (Westfall and Levin, 2018). In support of previous results, we found that loss of *crr* results in smaller cells (Figure 2, A and B); however, these cells are not as short or as thin as cells lacking *ptsI*.

Cell size has been linked to growth rate in what is termed the “Growth Law.” We measured the growth rates of strains deleted for the main PTS proteins *ptsI* (EI, PtsI) and *hpr* (PtsH) and found that they have a decreased growth rate compared with WT cells (Figure 1). Interestingly, while the *ptsI* complemented strain shows an increased length and width, it does not complement the growth rate defect (Figure 1). These results suggest that a fully functional PTS, specifically with a functioning glucose importer, is needed for proper cell size regulation and growth.

### The PTS acts independently of periplasmic glycans to regulate cell size

Previous work trying to connect nutrient availability and cell size found that UDP-glucose can act as a glucose sensor and regulate size through interaction with the division protein FtsZ (Chien *et al.*, 2012; Hill *et al.*, 2013). Deletion of *opgH*, the gene involved in the synthesis of periplasmic glucans that uses UDP-glucose as a substrate, results in short fat cells. To determine if the PTS works through this mechanism, we made an *opgH ptsI* double mutant. This mutant shows an additive effect of the two mutations, resulting in cells shorter than either single mutant and with an intermediate width (Supplemental Figure S2). This suggests that the PTS works through a different mechanism to regulate cell size than OpgH.

### cAMP is a major regulator of cell length

In the above experiments, cells were grown in LB medium with no added glucose. Why then is Crr, the glucose importer, necessary for proper cell size regulation? The phosphorylated state of Crr is used as a glucose sensor to activate catabolite repression. When glucose is present in the medium, it will be imported into the cell and phosphorylated by EII, resulting in an unphosphorylated EII. However, if there is no glucose, EII will be phosphorylated and can activate adenylate cyclase (CyaA) to produce cAMP. cAMP is an inducer of Crp, which can regulate a variety of genes. The loss of *ptsI*, *hpr*, and *crr* should mimic a high glucose situation (unphosphorylated EII), leading to low levels of cAMP; therefore, we deleted *cyaA* and *crp* to determine if the cell size changes in the *ptsI* mutant are due to a lack of cAMP or activated Crp, and measured cAMP levels in these cells.

As previously reported, deletion of either *crp* or *cyaA* results in cells that are smaller than WT (Figure 2, A and B; Westfall and Levin, 2018). Similar to loss of *crr*, we observed that loss of *crp* results in cells that are shorter and thinner than WT cells, but unlike *∆crr* whose cells are both longer and wider than *∆ptsI* the loss of *crp* does not lead to reproducible differences when compared with the *∆ptsI* cells (Figure 2, Data table). Interestingly, the *cyaA* mutant is shorter than the *ptsI* mutant. This suggests cAMP plays a large role in regulating cell length. The *cyaA* deletion strain is also shorter than the *crp* deletion strain, suggesting the cAMP or adenylate cyclase plays at least two roles in regulating cell length: 1) through the activation of Crp, and 2) through an unknown mechanism. Furthermore, while the *crr, cyaA,* and *crp* mutants are all the same width and thinner than WT, they are wider than the *ptsI* mutant, suggesting another mechanism for cell width regulation by the PTS that is independent of cAMP levels.

The loss of the PTS should mimic a high glucose state and lead to low levels of cAMP production. We measured cAMP levels in the *ptsI* deletion strain and confirmed that there is less cAMP than in WT cells. Complementation with a WT allele leads to an increase of cAMP over the deletion but not to WT levels, while complementation with the *ptsI_H189A_* allele did not result in increased cAMP production (Table 2). We would similarly expect a *crr* deletion to have low cAMP levels, as CyaA cannot be activated, and a *cyaA* deletion to have low levels of cAMP as synthesis is disrupted. As Crp represses *cyaA* expression, we would expect to observe increased cAMP in this mutant (Aiba, 1985). All three strains exhibited the expected cAMP phenotypes (Table 2). It has been previously shown that a *crr* deletion leads to a slower growth rate (Westfall and Levin, 2018). We therefore measured the growth of the *cyaA* and *crp* deletion strains. While both cells are smaller than WT, the *crp* deletion has a similar growth rate, while the *cyaA* mutant grows much slower (Supplemental Table S2), indicating that growth rate alone is not sufficient to decrease cell size.

To further determine if the cell size defects in all of these mutants are acting in a single pathway, we performed epistasis analysis by making double deletions of all four genes in all combinations. Mutant combinations lacking *cyaA* are short, supporting the idea of cAMP in length regulation (Figure 2C, Data Table). In further support of cell width being regulated by the PTS through a cAMP-CRP independent mechanism, we found that the *ptsI crp* and *ptsI cyaA* mutants exhibit an additive effect on cell width; these cells are thinner than either parent (Figure 2D, Data table). This suggests that there is an additional pathway acting on cell width that is perturbed in the *ptsI* mutant but not in the *cyaA* or *crp* mutants. While the loss of PTS regulation of CyaA likely results in changes to cAMP levels and therefore CRP activity, this would most likely not be as large as the deletion of *cyaA* or *crp,* as there is most likely some basal level of activity even in the absence of the PTS.

### Pyruvate/PEP regulates cell width

When cells are grown in a low sugar medium, the PTS system is fully phosphorylated so that it can rapidly act when sugar becomes available. We hypothesize that the other cell width regulator involves PEP or pyruvate, as PEP is the phosphate donor for EI. In the absence of *ptsI,* there may be a change in the PEP::pyruvate ratio in the cell as the PTS system is unable to use PEP (Long *et al.*, 2017).

**FIGURE 3:**
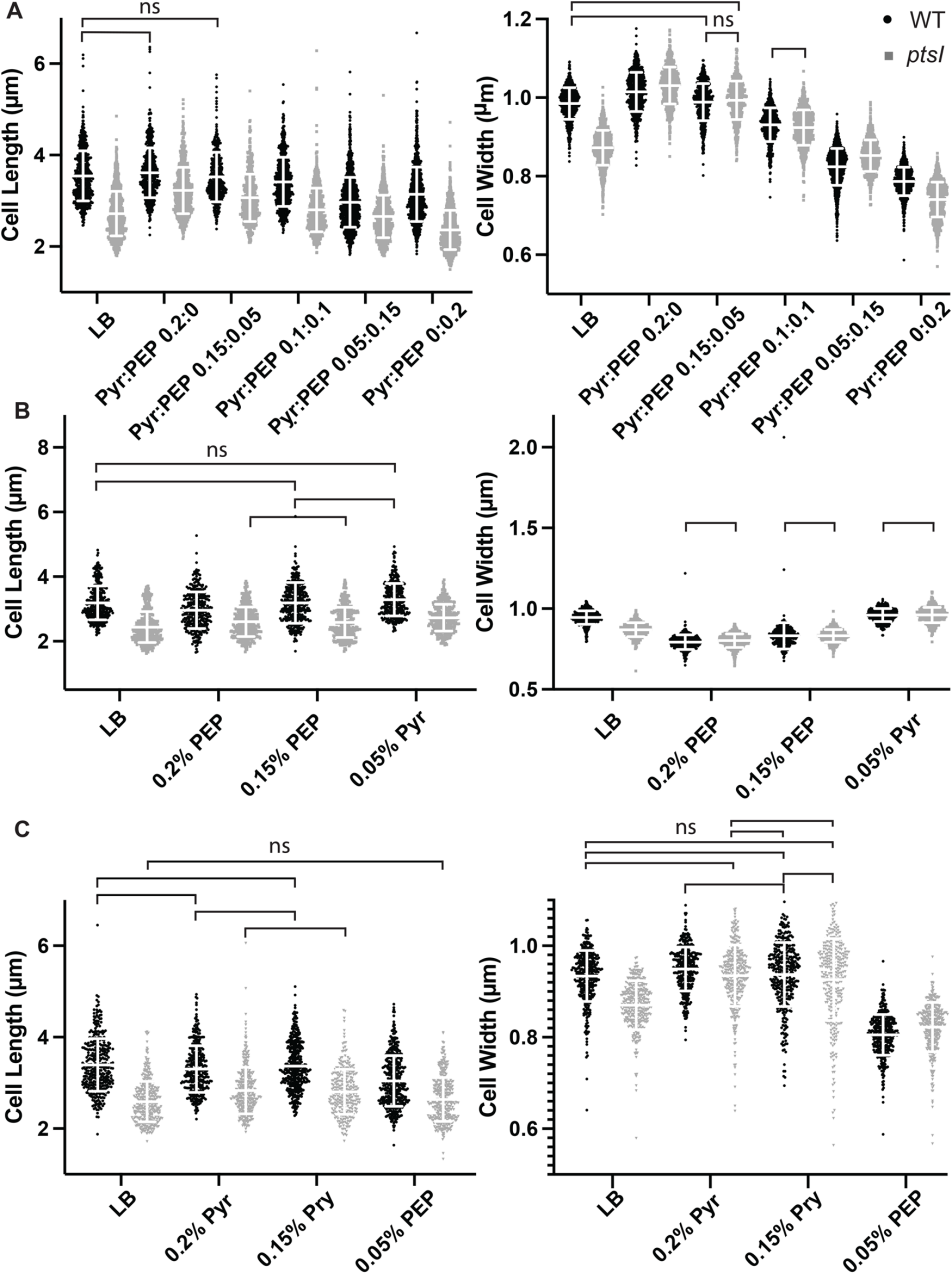
Pyruvate regulates cell width. WT or ∆*ptsI* cells were grown in LB medium supplemented with different amounts of PEP or pyruvate (pyr) to mid-log phase. Length and width measurements are shown. Pyruvate or PEP were added to the medium at a final concentration of 0.2%. Indicated comparisons are not statistically significant. Blue bars are mean with SD. A) Numbers indicate percentages. All comparisons are statistically significant from a one-way Anova and Tukey's multiple comparison test *p* < 0.001, unless noted. For length: *ptsI – ptsI* 0.1%:0.1%*, ptsI* 2%:0 *–* WT 0:2%, WT 0.15%:0.05% – WT 0.1%:0.1%, *ptsI* 0.15%:0.05% – WT 0.05%:0.15% have a *p* < 0.05. B) All comparisons are statistically significant from a one-way Anova and Tukey's multiple comparison test *p* < 0.001. For length: *ptsI*_LB_ – *ptsI*_0.15% PEP_ and *ptsI*_0.15% PEP_ – *ptsI*_0.05% Pyr_ have a *p* < 0.005 and *ptsI*_0.2% PEP_ – *ptsI*_0.05% Pyr_ has a *p* < 0.01. C) All comparisons are statistically significant from a one-way Anova and Tukey's multiple comparison test *p* < 0.001. For width: WT_0.05% PEP_ - *ptsI*_0.05% PEP_ has a *p* < 0.005 and WT_LB_ – WT_0.2% Pyr_ and WT_0.2% Pyr_ - *ptsI*_0.2% Pyr_ have a *p* < 0.05. For cell numbers, refer to Supplemental Table S3, and for images, refer to Supplemental Figure S4.

To test for a role of PEP or pyruvate in cell width regulation, cells were grown in LB media with different combinations of PEP and/or pyruvate added to the medium so that the total amount of them combined was 0.2%. When pyruvate is added alone (0.2%, 0.2:0), there is partial complementation of the length defect in a *ptsI* mutant, as we observed an increase in cell length (Figure 3, Supplemental Figure S3, Data table). Additionally, 0.2% pyruvate can fully complement the width defect of a *ptsI* mutant (Figure 3, A and C, Data table). Furthermore, the addition of 0.2% PEP (0:0.2) leads to a reduction in the cell width of both WT and ∆*ptsI* cells (Figure 3, A and B, Data table). When cells are grown in a combination of pyruvate and PEP, keeping the total carbon source levels at 0.2% cell width begins to reduce as PEP is added, and pyruvate is subtracted. This occurs even with 0.15% pyruvate and 0.05% PEP (0.15:0.05; Figure 3A, Data table).

To determine if the complementation of cell width by pyruvate was inhibited by the low levels of added PEP or because the needed amount of pyruvate was not high enough, we tested the individual concentrations of PEP and pyruvate used in the mixed experiments (Figure 3, B and C). Small amounts of PEP or pyruvate are able to affect cell width, as 0.05% pyruvate leads to an increase in cell width to a similar level as 0.2% pyruvate in the ∆*ptsI* strain, and 0.05% PEP leads to a reduction of cell width in both strains, similar to 0.2% PEP. These data suggest that the cell is responding to changes in the intracellular ratio of PEP and pyruvate.

As there is no known PEP importer, we tested whether WT or ∆*ptsI* cells are able to grow with PEP as the sole carbon source. While there is a distinct growth rate difference between growth on glucose or PEP for WT cells, both strains are able to grow with PEP as the sole carbon source (Supplemental Figure S3). As both PEP and pyruvate can be used as carbon sources by the cell, changes in cell size should not be directly due to nutrient availability. In a WT cell, changes to PEP::pyruvate levels may affect the phosphorylation state of Crr (Hogema *et al.*, 1998). Additional PEP in a WT cell may mimic the conditions of the *ptsI* mutant, as without EI to use PEP, we propose the levels would increase in the cell.

### Pyruvate and PEP affect cell growth

As cell size is related to growth rate when nutrients are changed, we measured the growth rate of WT and ∆*ptsI* cells in LB (Figure 1A; Table 1). As expected, based on the reduced size of the *ptsI* mutant, these cells also have a decreased growth rate. As the addition of PEP or pyruvate can alter the cell size, we also measured growth in different combinations of added pyruvate and PEP (as in Figure 3A). The addition of pyruvate to the medium can complement the growth defect of the *ptsI* mutant. Interestingly, as PEP is added (or pyruvate removed) to the media, both WT and ∆*ptsI* cells show a reduction in growth rate. While there is only a slight decrease in the growth rate of WT cells from LB to the 1:1 (0.1% each) condition, there is a dramatic reduction in cell width (0.99 to 0.93). These data suggest that while there is a connection between the PTS, cell size, and growth rate, there is not a direct correlation between growth rate and cell size due to changes in PEP or pyruvate levels. It is also interesting that growth in PEP reduces growth rate, even though this is a viable carbon source for the cells (Supplemental Figure S3).

**TABLE 1:** Max growth rate of strains in media supplemented with differing amounts of pyruvate or PEP.

	LB	Pyr:PEP 0.2%:0%	Pyr:PEP 0.15%:0.05%	Pyr:PEP 0.1%:0.1%	Pyr:PEP 0.05%:0.15%	Pyr:PEP 0%:0.2%
WT	28.7	28.5	29.3	29.8	34.1	39.3
*ptsI*	36.2	27.7	32.2	38.2	37.2	40.8

**FIGURE 4:**
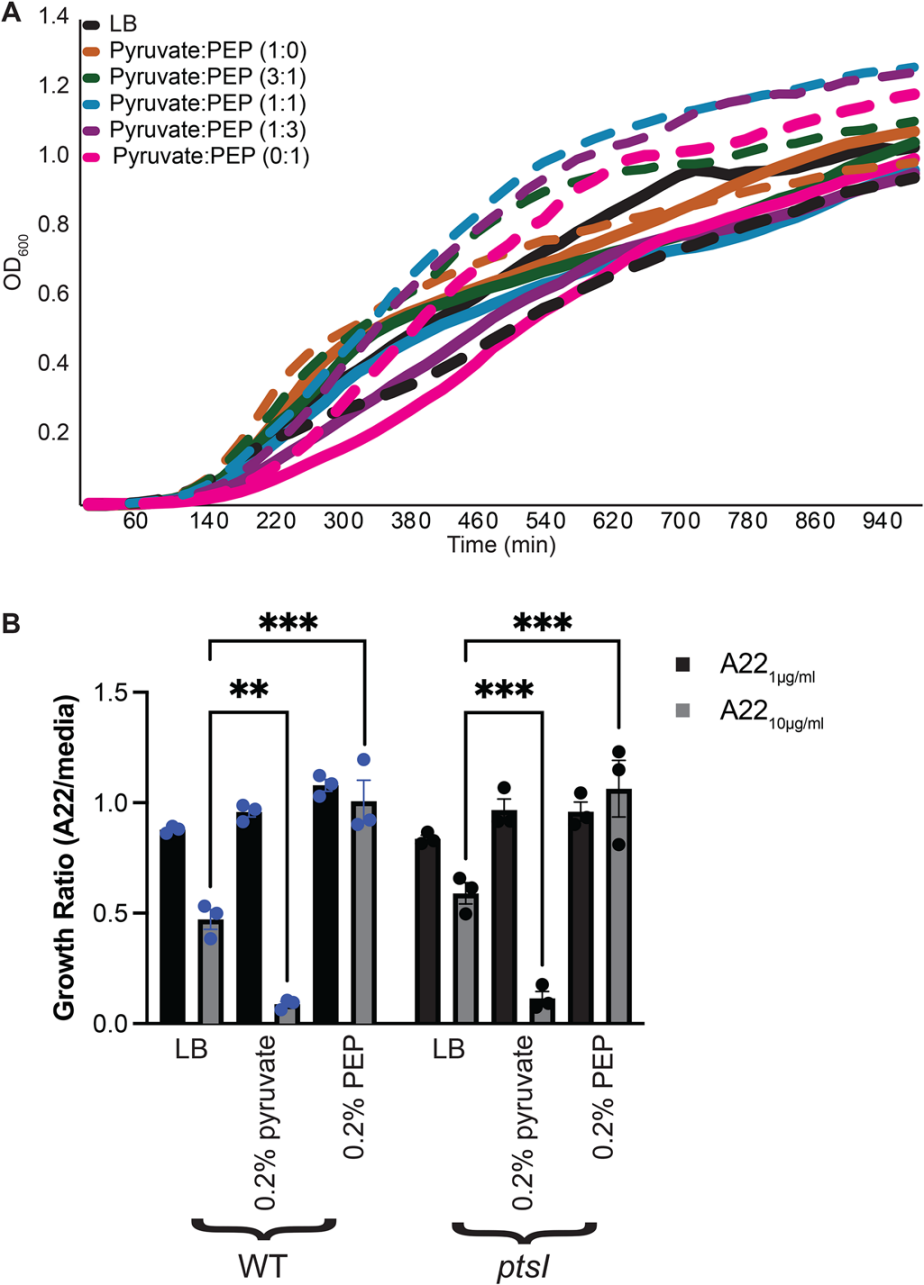
The addition of pyruvate and PEP affect growth and A22 resistance. A) Growth curve of WT and *∆ptsI* cells grown in LB or LB supplemented with the same combinations of pyruvate and PEP from Figure 3A. Cells were grown overnight in 2 ml cultures before being diluted to an OD of 1.0. 0.5 µl were inoculated into a 96-well plate filled with 150 µl of each media with six technical replicates of each strain per condition. Shown is a representative growth curve averaging the six replicates from one experiment. The trends shown here are reproducible over three independent biological replicates. Refer to Table 1 for the average growth rate. Solid line are WT and dashed lines are ∆*ptsI* cells grown in the indicated conditions. B) Cells were grown overnight in 2 ml tubes and then diluted 1:1000 into fresh LB or LB + A22 tubes and grown shaking at 37°C for 6 h. The ratio of the OD from the LB vs A22 tubes is shown. This is an average of three independent experiments. Error bars are SD. Statistical significance was determined by two-way Anova and Tukey's multiple comparison test ** *p* < 0.005, *** *p* < 0.001.

We observed that ∆*ptsI* cells have a similar maximum growth rate as WT cells when grown with the addition of PEP either alone, where both strains grow slowly, or in mixtures with pyruvate (Table 1). This occurs in the early log phase. As the cells continue to grow, there is a separation in the growth curves, and it appears that the ∆*ptsI* cells grow better than WT (Figure 4A). This is clearest when looking at cells grown in 1:1 (0.1% each), 1:3 (0.05 Pyr and 0.15% PEP), or 0:1 (0.2% PEP) ratios. When the media is supplemented with pyruvate, the two growth curves are more similar (brown lines, Figure 4A). These results align with our previous result that when PEP is the sole carbon source, ∆ *ptsI* cells grow better than WT cells (Supplemental Figure S3).

### Pyruvate and PEP affect A22 resistance

Our initial observation was that the loss of *ptsI* suppresses the A22 sensitivity of an *envC* deletion and that this resistance extended to WT cells (Supplemental Figure S1; Sloan *et al.*, 2022). Therefore, we examined the effects of adding 0.2% pyruvate or PEP on A22 sensitivity. The addition of pyruvate leads to a dramatic increase in sensitivity to A22 in both WT and ∆*ptsI* cells, while the addition of PEP leads to an increase in A22 resistance (Figure 4B). Slow growth is a known suppressor of an *mreB* deletion, so it might be that changes in growth rate caused by the addition of pyruvate or PEP to the media are the reason for the changes to A22 resistance (Bendezú and De Boer, 2008). We do not believe this to be the case for multiple reasons. 1) The slow growth phenotype observed as a suppressor was much more pronounced than we see for ∆*ptsI* cells, even after the addition of PEP, and 2) the increased sensitivity to the addition of pyruvate is seen in both WT and ∆*ptsI* cells, with no change in growth rate observed for the WT cells. This suggests that the effect of pyruvate and most likely PEP on changes to A22 sensitivity is independent of changes to growth rate. These results further connect the metabolic state of the cell to cell size regulation by showing that changes to metabolites can alter how cells respond to the disruption of a major cell shape determinant, MreB.

### Pyruvate metabolism is important for cell size regulation

**FIGURE 5:**
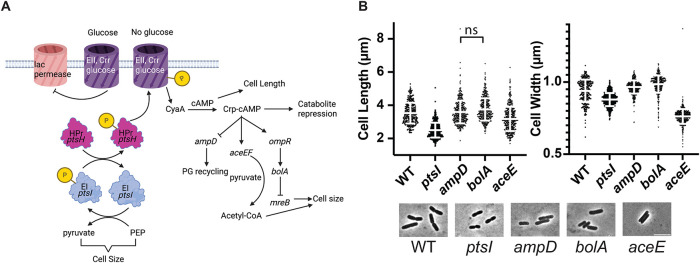
Pyruvate dehydrogenase is important for cell shape regulation. A) Model showing the regulation of the PTS and selected genes in the Crp regulon (Created in https://BioRender.com). Arrows indicate activation and bars show repression. AceEF convert pyruvate to acetyl-CoA. B) Cells were grown in LB media to mid-log phase. Length and width measurements of indicated strains are shown. All comparisons are statistically significant from a one-way Anova and Tukey's multiple comparison test *p* < 0.001, unless indicated. ns = not significant. Blue bars are mean with SD. *N* > 450.

The *ptsI crp* double mutant shows an additive effect of cell thinning (Figure 2, E and F), suggesting that there are two pathways involved in width regulation. To determine how Crp regulates cell width, we examined the Crp regulon for genes that could play a role in cell size regulation (Figure 5A). Phosphorylated Crr activates adenylate cyclase to produce cAMP, which then can act on a variety of genes as either a repressor or activator. BolA is a known repressor of MreB that has been suggested to be the cAMP-regulated gene that is responsible for cell shape changes (Lange et al., 1991; Freire et al., 2009; Westfall and Levin, 2018). In addition to *bolA,* we identified *ampD* and *aceE* as possible genes involved in cell width regulation. AmpD is involved in cell wall recycling and is repressed by Crp-cAMP, while AceE is part of the pyruvate dehydrogenase complex responsible for converting pyruvate into acetyl-CoA and is activated by Crp-cAMP (Figure 5A).

To determine if any of these genes influence cell size, we measured the size of cells deleted for each gene. BolA is a stationary phase protein that inhibits *mreB* expression. Overexpression of *bolA* during the exponential phase has been shown to reduce cell size (Freire *et al.*, 2009; Lange and Hengge-Aronis, 1991). As expected, because *bolA* is not normally expressed during the exponential phase, its deletion does not have a large effect on cell size. Similarly, loss of *ampD* does not have a large influence on cell size. We did see a drastic effect of the *aceE* deletion on both length and width, with the largest change occurring for cell width, similar to previous results, which also showed a reduced growth rate (Figure 5B; Westfall and Levin, 2018). Interestingly, loss of *aceE* also leads to lower levels of cAMP (Table 2). This suggests that there are two inputs into pyruvate levels that are disrupted when *ptsI* is deleted. First, there is the loss of the direct production of pyruvate from PEP. Second, there is a change in the breakdown of pyruvate into acetyl-CoA by AceE due to changes in Crp activity from changes in cAMP production based on changes in the phosphorylation state of EII.

**TABLE 2:** Relative intracellular cAMP levels.

Strain	Relative cAMP/O.D.
MG1655 + pBad	++
∆*ptsI* + pBad	−
∆*ptsH* + pBad	++
∆*ptsI* + *ptsI_WT_*	+
∆*ptsI* + *ptsI_H189A_*	−
MG1655	++
∆*cyaA*	−
∆*crr*	−
∆*crp*	+++
∆*aceE*	−

### Exogenous fatty acids affect cell width

Changes in acetyl-CoA levels due to loss of *aceE* or changes in the cellular pyruvate pool can lead to changes in fatty acid synthesis. Inhibition of fatty acid synthesis has been shown to affect cell size (Westfall and Levin, 2018; Yao *et al.*, 2012). To determine if the observed cell size defects in a *ptsI* mutant are due to a lack of fatty acid synthesis, we grew WT and ∆*ptsI* cells in LB or LB supplemented with the long-chain fatty acid, oleic acid. While oleic acid does not affect the length of either WT or ∆*ptsI*, it is able to increase the width of WT cells by 0.05 µm (*p* < 0.0001) and ∆*ptsI* cells by 0.064 µm (*p* < 0.0001; Supplemental Figure S5). The fact that the addition of fatty acid to the media causes WT cells to increase in width suggests that this phenomenon is independent of perturbed fatty acid synthesis in the *ptsI* mutant and therefore suggests that the role of pyruvate in regulating cell size is not through acetyl-CoA production and fatty acid synthesis. We have previously suggested that changes in PEP or pyruvate levels could lead to changes in the synthesis of cell wall precursors (Barton *et al.*, 2021). It is possible that changes in precursor levels have an effect on the cell size and A22 sensitivity changes seen in *ptsI* deletion cells.

## DISCUSSION

We show that loss of the conserved proteins in the PTS, Enzyme I and HPr, regulate both cell width and length through two mechanisms. The first mechanism regulates length through the phosphorylation of the Enzyme II for glucose (Crr) and its regulation of cAMP production (Figure 2). The second mechanism regulates width through changes to the PEP::pyruvate (Figure 3). These mechanisms are independent of OpgH, suggesting a potentially new connection between metabolism and cell size regulation (Supplemental Figure S2).

This work supports previous work that has shown changes to cAMP levels influence cell size (Westfall and Levin, 2018). In this previous report, cAMP was suggested to have a large effect on width, not length, as we see here; however, this previous study and the work presented here both conclude that cAMP works through two pathways to regulate cell size. The first is through catabolite repression and the activation of Crp with a second unknown mechanism (Figure 2). Different roles for Crp-cAMP have been proposed. One mechanism involves the stationary phase morphogene, BolA, which represses *mreB* expression. Deletion of this gene has only a minor effect on exponential cell size (Figure 5B), although it is possible that changes in catabolite repression lead to its overexpression during the exponential phase, resulting in smaller cells (Westfall and Levin, 2018). A second proposed mechanism for Crp-cAMP cell size regulation works through pyruvate dehydrogenase (AceE). Cells lacking cAMP or *crp* would not be able to induce expression of *aceE*, possibly leading to changes in pyruvate levels (Figure 5). This may explain why there is a slight reduction of the *crr*, *crp*, and *cyaA* mutants, as we have proposed that changes to pyruvate regulate cell width. As loss of *aceE* leads to less cAMP, this could explain the reduced length of this mutant and help to reinforce any other *crp* phenotypes.

When *ptsI* is missing from the cell, the entire PTS is locked in an unphosphorylated state. This state mimics a high glucose condition, as when glucose is present, the phosphates from the PTS (Crr) are constantly transferred to glucose. An unphosphorylated EII would not activate the production of cAMP, leading to low levels of cAMP (Table 2) and low levels of Crp activation. It is interesting that cells lacking the PTS are small as cells are larger when grown in the presence of glucose and therefore have low cAMP. With Crp stuck in an inactive state in the PTS mutants, the levels of *aceE* should remain low (and *bolA* would possibly increase), leading to less degradation of pyruvate. This increase in pyruvate may be countered by the decrease in PEP dephosphorylation. The loss of EI would change the ratio of PEP and pyruvate, as PEP is the phosphodonor for the system. In total, the effects of cAMP (or adenylate cyclase) through its activation of Crp and an unknown pathway would be minimized, and pyruvate levels would change, causing changes to both the length and width of cells. Consistent with these results, it has been previously shown that changes to the PEP-pyruvate ratio can affect catabolite repression through changes in Crr phosphorylation (Hogema *et al.*, 1998). Furthermore, while the loss of *aceE* should result in increased pyruvate levels, it also grows slowly (Westfall and Levin, 2018). This slow-growth small-cell phenotype appears to be dominant to the increase in size seen by the addition of pyruvate to the medium (Figure 3). It is quite interesting that the addition of PEP to the media has the opposite effect as pyruvate, as PEP and pyruvate act as usable carbon sources (Supplemental Figure S3). The fact that the addition of these leads to opposite cell size phenotypes suggests that PEP::pyruvate acts as a signal to activate or repress other genes involved in cell size control (Figure 3). If PEP levels increase, the cell glycolysis is blocked by the allosteric inhibition of PfkA, leading to increased phosphorylation of Crr, leading to increased cAMP levels (Shimizu, 2013, 2016).

There are three pyruvate importers in *E. coli*: *btsT, cstA,* and *yhjX* (Hwang *et al*., 2018). The former two genes are positively regulated by Crp-cAMP (Blum *et al.*, 1990; Gasperotti *et al.*, 2020; Schultz and Matin, 1991), while the latter is controlled by the two-component system, PyrSR, which is activated by pyruvate (Miyake *et al.*, 2019). Induction of *btsT* is also controlled by the pyruvate and nutrient-sensing BtsSR two-component system. In addition, these importers are regulated by the global metabolic regulator CsrA (Behr *et al.*, 2014; Dubey *et al.*, 2003). The inability to activate carbon utilization genes in cells with low cAMP due to the lack of a PTS could lead to starvation-like conditions as the cells use the available carbon sources in the media.

It is possible that changes in cell size due to the addition of pyruvate to the media are not caused by metabolic changes but rather changes in the expression of other genes controlled by these systems, such as *pbpC* (PBP1C)*,* a minor cell wall synthesis gene regulated by PyrSR (Miyake *et al.*, 2019). Cell size in most bacteria is controlled by the synthesis of the cell envelope. The cell wall is thought to provide the shape, as removal of the wall leads to a loss of rod shape. During normal growth, *E. coli* cell shape is regulated by the MreB-controlled elongasome complex. The major cell wall synthesis enzymes RodA and PBP2 build the wall. While loss of *rodA* or *mrdA* (PBP2) is lethal, there is no effect on growth rate or sensitivity to cell wall targeting antibiotics in a *pbpC* deletion (Schiffer and Höltje, 1999). If changes to pyruvate or PEP regulate the expression of cell wall synthesis enzymes that are only used during certain carbon limitations, this would provide a new link between cell size and metabolism and better define roles for the minor PG synthases. While *mreB* mutations have been shown to regulate cell size along with rod shape, a mechanism for this is unknown (Maharjan *et al.*, 2026; Ouzounov *et al.*, 2016). It is currently unclear if changes in size due to MreB mutations are related to changes in size due to metabolic changes. One possibility is that MreB interacts with PBP1C, which regulates cell wall synthesis due to pyruvate levels.

Additionally, we noticed that the addition of pyruvate (0.2%:0%) leads to very similar growth rates between WT and ∆*ptsI* cells with similarly shaped growth curves. The addition of PEP (0%:0.2%) also leads to similar max growth rates, but not similarly shaped growth curves. The max growth rate is observed in the early exponential phase (∼T120), but later the curves separate. It is unclear if PEP itself is slightly toxic to the cells or if PEP is blocking the use of alternative carbon sources. Cells normally progress through different amino acids in LB as their carbon source, so the similar max growth rates may be due to eating a shared carbon source, but ∆*ptsI* cells can utilize PEP better than WT and therefore grow better once the early carbon sources are metabolized.

These results further add to our knowledge of how cell size is regulated by metabolism. We have supported past findings that cAMP is a major cell size regulator and that it most likely works through two pathways. We have further shown that pyruvate is a major metabolite in the regulation of cell size. Future work will be needed to understand how pyruvate levels regulate cell width and the second pathway for cAMP-regulated cell size changes.

## MATERIALS AND METHODS

Request a protocol through *Bio-protocol*

### Bacterial growth

Bacteria were grown using standard laboratory conditions. In total, 2 ml cultures were grown overnight in LB medium (10 g/l NaCl, 10 g/l tryptone, 5 g/l yeast extract) and subcultured in the morning 1:1000 into tubes with 2 ml of media and grown to exponential phase (O.D._600_ 0.3–0.6) at 37°C in a shaking incubator. Keio collection mutants were moved into MG1655 using P1 transduction. Transductants were selected on kanamycin (30 µg/ml) and confirmed by PCR. Media was supplemented with carbon sources as indicated (Sodium Pyruvate (ThermoScientific), Phosphoenolpyruvic acid monopotassium salt (Alfa Aesar). See Supplemental Table S1 for a list of strains used in this study.

### Growth curves and rates

Cultures were grown overnight in appropriate antibiotics in LB. For LB growth curves, cells were inoculated into a 96-well plate at an O.D. of 0.0033 with six replicates per strain. Instantaneous growth rates were determined by using a sliding window of 80 min around each time point to fit an exponential growth model. The max growth rate is reported.

For minimal media growth curves, cells were grown overnight in LB. Cells were diluted to an O.D. of 1.0 in PBS and inoculated into a 96-well plate at an O.D. of 0.0033 with 12 replicates per strain per media. Cells were grown in M63 media with no case amino acids or thiamine added. PEP or glucose was added at 0.2%.

### Double mutant construction

Single mutants were made via transduction from the Keio collection. For the production of double mutants, single mutants were transformed with pCP20 encoding a flippase to remove the kanamycin resistance gene from the single mutants, leaving an frt scar. After curing of the plasmid with growth at 42°C, the cells were transduced with the second mutation and selected on kanamycin.

### Microscopy

For all imaging, cells were grown at 37°C in LB medium until exponential phase (0.3–0.6 OD_600_). We attempted to image all cells for any single experiment on the same day at the same OD. Samples and their replicates were imaged within 0.5 OD of each other. All imaging data represent pooled data from three independent biological replicates collected on different days. At least 100 cells were collected for each sample in each replicate.

Imaging was performed on 1% M63-glucose agarose pads at room temperature. Phase contrast images were collected on a Nikon Ni-E epifluorescent microscope equipped with a 100X/1.45 NA objective (Nikon), Zyla 4.2 plus cooled sCMOS camera (Andor), and NIS Elements software (Nikon).

Cell size was calculated using the MATLAB software Morphometrics (Ursell *et al.*, 2017) and custom software as described previously. Only non-dividing cells were used for analysis unless stated otherwise.

### A22 assays

Overnight cultures were subcultured 1:1000 into fresh media supplemented with different concentrations of A22. After six hours of shaking growth at 37°C the OD_600_ was measured. The ratio of ODs between the LB-only control and A22 tube is used to determine if cells are more or less sensitive to the drug.

### cAMP measurement

Intracellular cAMP was measured via an ELISA (Cayman Chemical 581001). In total, 1 ml of cells were grown in LB to mid-log phase and washed twice in PBS. Cells were resuspended in 0.5 ml of PBS and sonicated on ice at 70% power with 15 pulses.

## Supporting information







## Abbreviations used:

ATP: adenosine triphosphate
cAMP: cyclic AMP
EI: enzyme 1 of the PTS
EII: enzyme 2 of the PTS
LB: Luria Bertani media
PEP: phosphoenolpyruvate
ppGpp: guanosine tetraphosphate
PTS: phosphoenolpyruvate phosphotransferase system
Pyr: pyruvate
UDP: uridine diphosphate
WT: wild type.
