# Supplementary material for: The phosphoenolpyruvate phosphotransferase system regulates cell length and width through semi-independent pathways
Source: Mol Biol Cell. 2026 Jul 27;37(8):ar77. doi: 10.1091/mbc.E26-03-0129 (PMC13426487; doi:10.1091/mbc.E26-03-0129)
Supplement: Supplementary file 1 [file mbc-37-ar77-s001.pdf]

Supplemental Materials

Molecular Biology of the Cell

Surber *et al*.

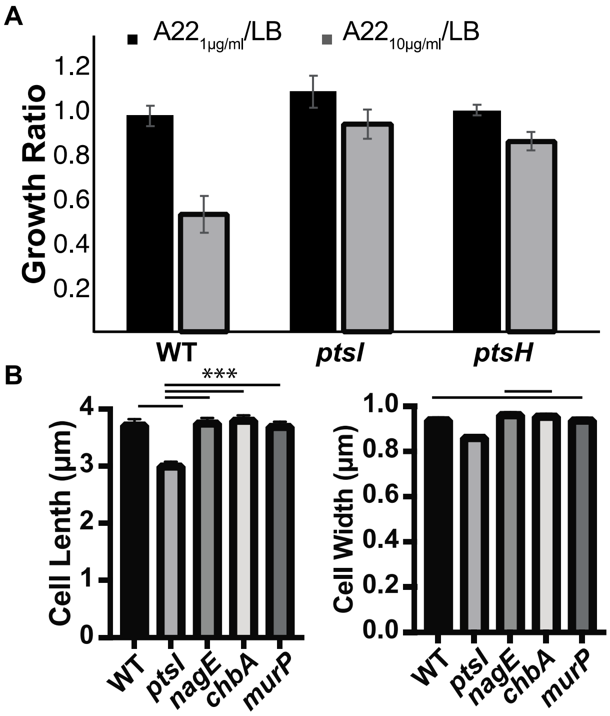

Figure S1: An intact PTS is needed for proper cell size regulation. A) Indicated strains were grown in LB or LB supplemented with the indicated amount of A22. Cells were grown for six hours shaking at 37^{0}C. The OD_{600} of each strain grown in A22 was compared to that strain grown in LB. B) Cells were grown in LB media to mid-log phase. Length and width measurements of indicated strains are shown. Error bars are 95% CI. For length, if not indicated all interactions are not significant, *** = p <0.001. For width all comparisons not indicated are significant, p <0.001.

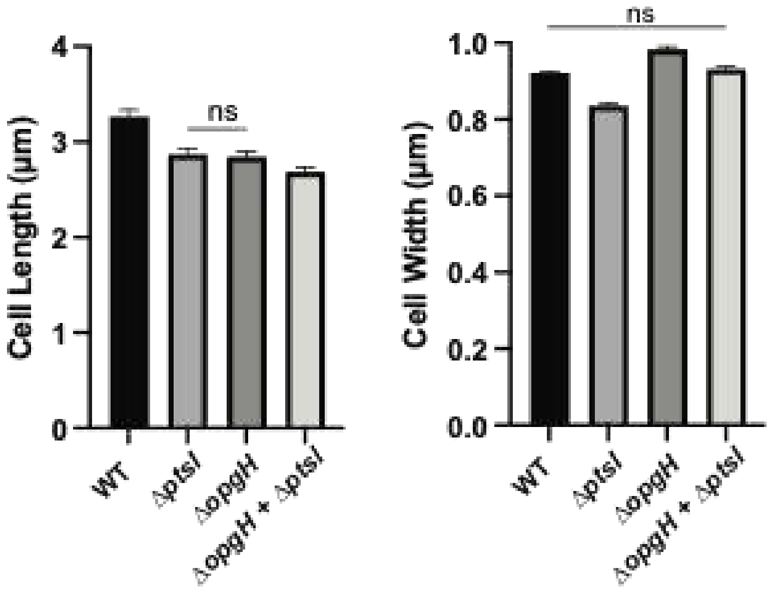

Figure S2. PTS cell size regulation is independent from OpgH. Cells were grown in LB media to mid-log phase. Length and width measurements of indicated strains are shown. Error bars are 95% CI. ns = not significant. All interactions are significant unless otherwise noted.

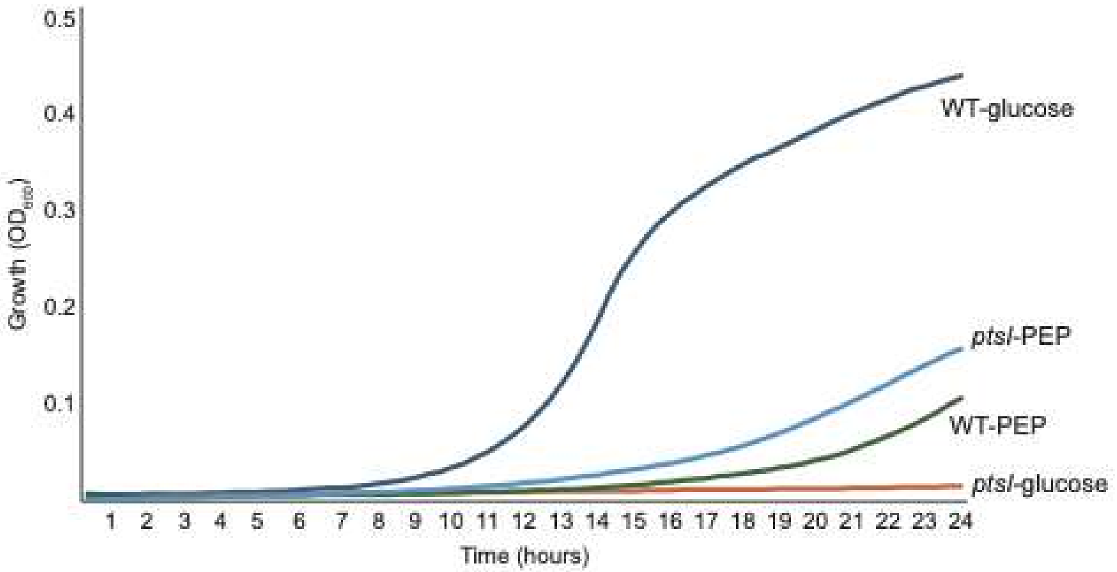

Figure S3. PEP is a viable carbon source. WT and Δ*ptsI* were grown in a 96 well plate in M63 minimal media with the indicated carbon source for 24 hours.

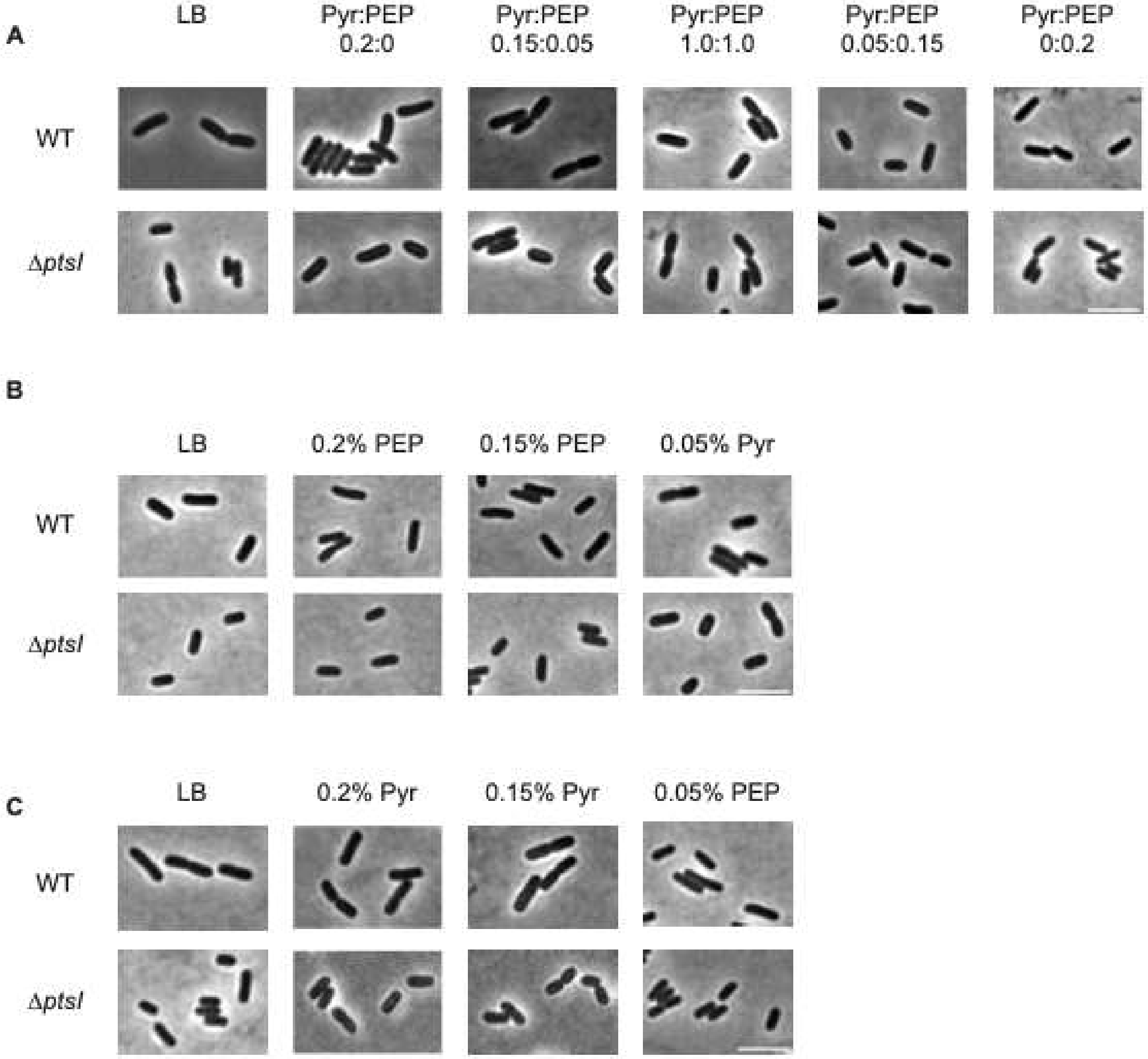

Figure S4. Images of cells grown in variable amounts of PEP and/or pyruvate. Representative images of data from Fig. 3. Scale bar = 5 μm.

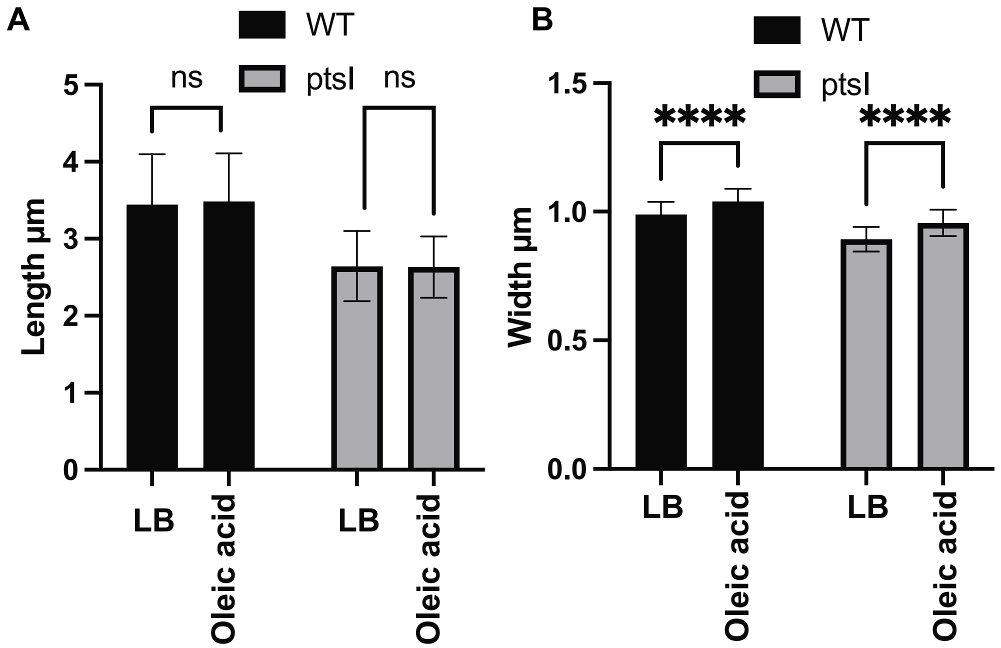

Figure S5. Inhibition of fatty acid synthesis is not the cause of cell size changes in PTS mutants. Cells were grown in LB or LB supplemented with 10 µg/ml of oleic acid to mid-log phase. Length and width measurements of indicated strains are shown. Error bars are 95% CI. ns = not significant. **** $p < 0.0001$.

Strain	Relevant Genotype	Source
MG1655	WT	Coli Stock Center
MG1655 + pBad18		
EH1	*ptsI*::kan	(*1*)
JS1	*chbA*::kan	This study
JS2	*nagE*::kan	This study
JS4	*opgH*::kan	This study
JS5	*ptsI*::frt, *opgH*::kan	This study
JS6	*cyaA*::kan	This study
JS7	*crr*::kan	This study
JS8	*crp*::kan	This study
JS9	*cyaA*::frt, *crr*::kan	This study
JS12	*cyaA*::frt, *crp*::kan	This study
JS15	*ptsI*::frt, *crp*::kan	This study
JS19	*crr*::frt, *crp*::kan	This study
JS20	*ampD*::kan	This study
AG26	*murP*::kan	(*2*)
RM555	*bolA*::kan	This study
RM610	*ptsH::kan*	(*1*)
RM627	pBad18-$ptsI_{H189A}$; *ptsI*::kan	(*3*)
RM628	pBad18-*ptsI*; *ptsI*::kan	(*3*)
RM664	*pts*I::frt, *crr*::kan	This study
RM665	*ptsI*::frt, *cya*::kan	This study
aceE	*aceE*::kan	(*4*)

Table S1: List of strains

1. R. Sloan, J. Surber, E. J. Roy, E. Hartig, R. M. Morgenstein, Enzyme 1 of the Phosphoenolpyruvate:sugar phosphotransferase System is Involved in Resistance to MreB Disruption in Wild-type and Δen*vC C*ells. *Mol Microbiol*, (2022).
2. A. Grinnell, R. Sloan, R. M. Morgenstein, Cell Density-dependent Antibiotic Tolerance to Inhibition of the Elongation Machinery Requires Fully Functional PBP1B. *Commun Biol* **5**, 107 (2022).
3. L. Lopian, Y. Elisha, A. Nussbaum-Shochat, O. Amster-Choder, Spatial and Temporal Organization of the *E. coli* PTS Components. *The EMBO Journal* **29**, 3630-3645 (2010).
4. C. S. Westfall, P. A. Levin, Comprehensive analysis of central carbon metabolism illuminates connections between nutrient availability, growth rate, and cell morphology in Escherichia coli. *PLOS Genetics* **14**, e1007205 (2018).

Table S2: Growth rate of strains in LB

Strain	Doubling time (min)
MG1655	23.3
Δ*crp*	25.9
Δ*cyaA*	33.5

Table S3. Cell numbers from figure 3.

	LB	Pyr:PEP 0.2%:0%	Pyr:PEP 0.15%:0.05%	Pyr:PEP 0.1%:0.1%	Pyr:PEP 0.05%:0.15%	Pyr:PEP 0%:0.2%
WT	638	598	499	657	1237	988
ptsI	1204	744	523	870	859	1046
	LB	.2% PEP	0.15% PEP	0.05% Pyr		
WT	410	355	394	339		
ptsI	422	428	375	281		
	LB	0.2% Pyr	0.15% Pyr	0.05% PEP		
WT	377	356	371	412		
ptsI	379	358	316	388		
